# Supplementary material for: Broad-Spectrum Antimicrobial Activity of Ultrafine (BiO)2CO3 NPs Functionalized with PVP That Can Overcome the Resistance to Ciprofloxacin, AgNPs and Meropenem in Pseudomonas aeruginosa
Source: Antibiotics (Basel). 2023 Apr 14;12(4):753. doi: 10.3390/antibiotics12040753 (PMC10135073; doi:10.3390/antibiotics12040753)
Supplement: Supplementary file 1 [file antibiotics-12-00753-s001.zip › antibiotics-2327908-supplementary.pdf]

Supplementary Information

Broad-spectrum antimicrobial activity of ultrafine $(BiO)_2CO_3$ NPs that can overcome the resistance of ciprofloxacin, AgNPs and meropenem in *P. aeruginosa*

Bishnu D. Pant, Nalin Abeydeera, Rabindra Dubadi, Min-Ho Kim, Songping D. Huang

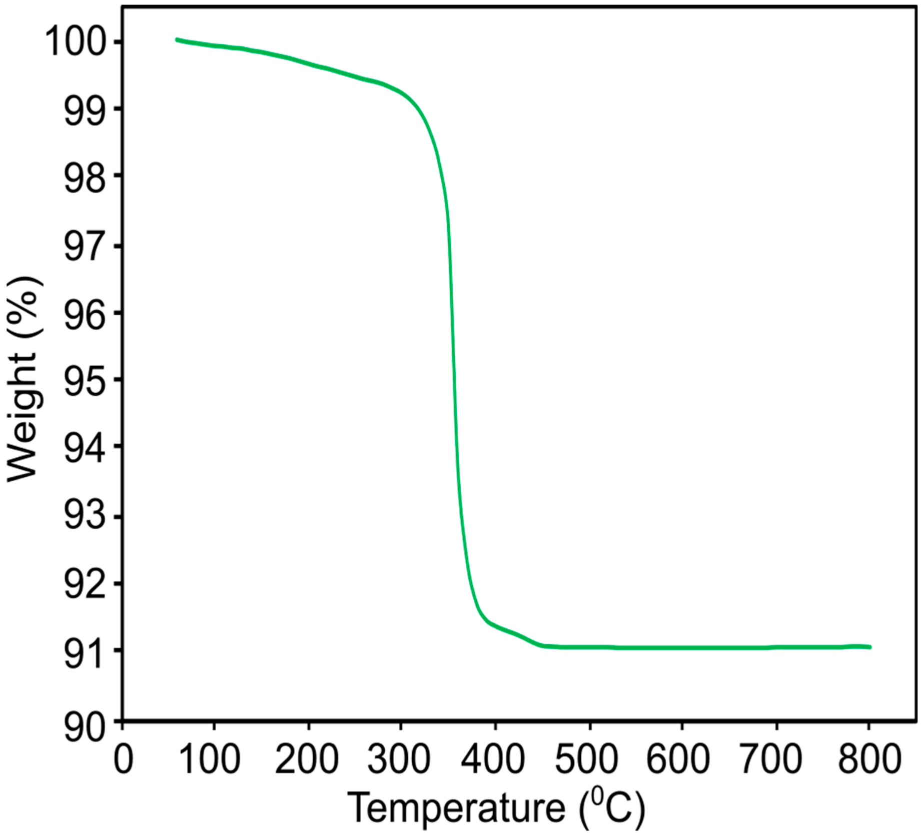

Figure S1 TGA curve of the as-synthesized bulk $(BiO)_2CO_3$ sample

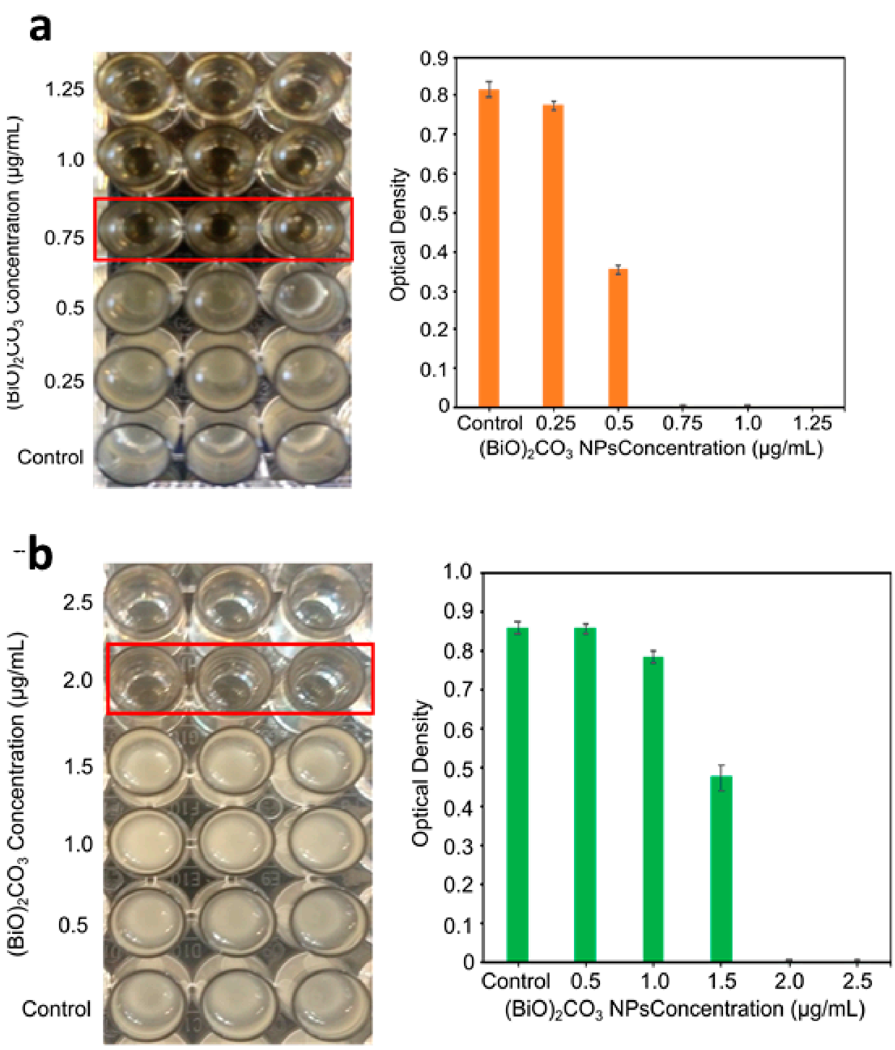

Figure S2 Representative images and plots of optical density value *vs*. concentration of $(BiO)_2CO_3$ NPs against (**a**) PA and (**b**) SA

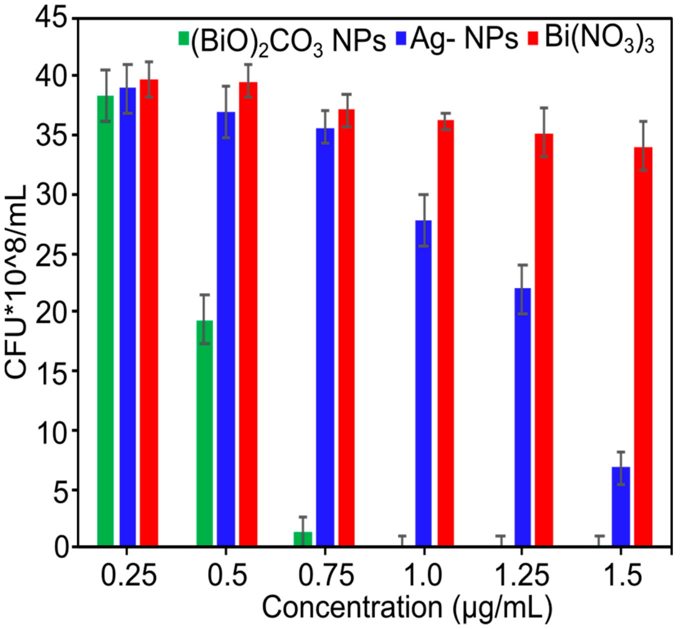

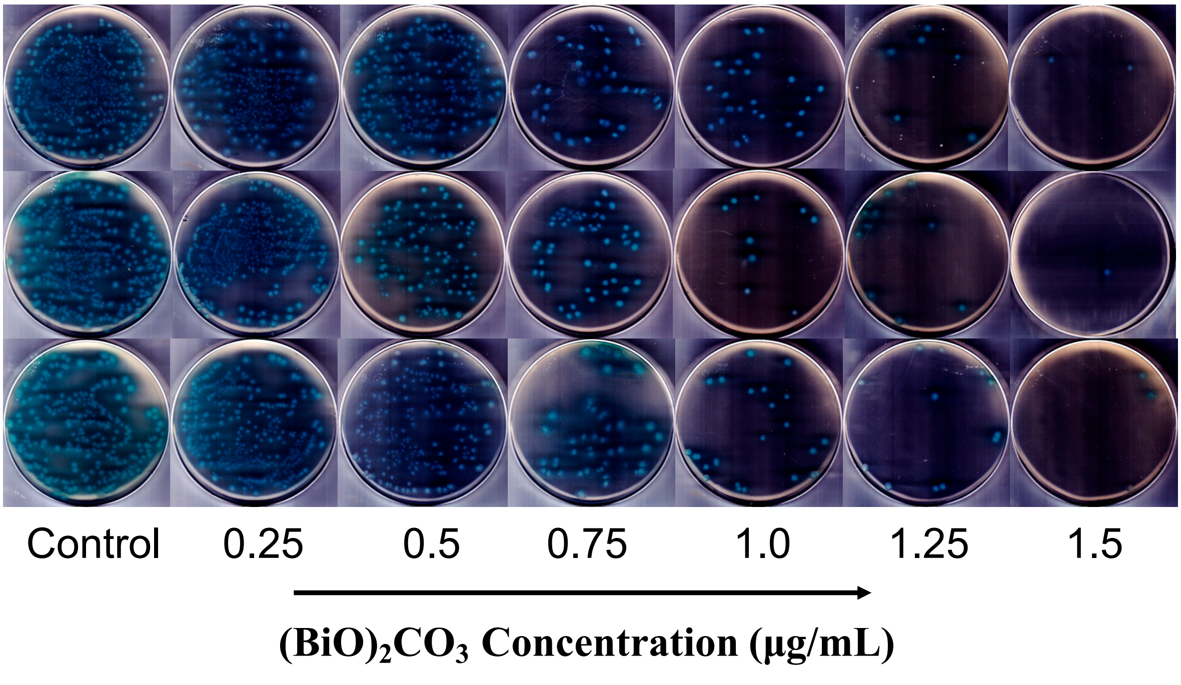

Figure S3 A representative plot of CFU enumeration results of $(BiO)_2CO_3$ NPs against PA and representative images of CFU enumeration results of $(BiO)_2CO_3$ NPs against PA

Table S1 MIC values of $(BiO)_2CO_3$ NPs against the AgNPs-, ciprofloxacin-, and meropenem-resistant PA for 30 consecutive days.

MIC Values							
**Day**	PA^{AgR}	PA^{cipR}	PA^{meroR}	**Day**	PA^{AgR}	PA^{cipR}	PA^{meroR}
**1**	1.25	1	1.75	**16**	1.5	1.25	5
**2**	1.25	1	1.75	**17**	1.5	1.25	2.5
**3**	1.25	1	1.75	**18**	1.25	1.25	5
**4**	1.25	1	1.75	**19**	1.25	1.25	5
**5**	1.25	1.25	1.75	**20**	1.25	1.25	5
**6**	1.25	1	1.75	**21**	1.25	1.25	5
**7**	1.5	1	2.5	**22**	1.25	1.25	5
**8**	1.5	1	2.5	**23**	1.5	1.25	10
**9**	1.25	1.25	2.5	**24**	1.5	1.25	10
**10**	1.25	1.25	2.5	**25**	1.75	1.5	10
**11**	1.25	1	5	**26**	1.75	1.25	10
**12**	1.25	1	5	**27**	2	1.25	20
**13**	1.5	1.25	2.5	**28**	2	1.5	10
**14**	1.5	1.25	2.5	**29**	4	1.5	20
**15**	1.5	1.25	5	**30**	4	1.5	20

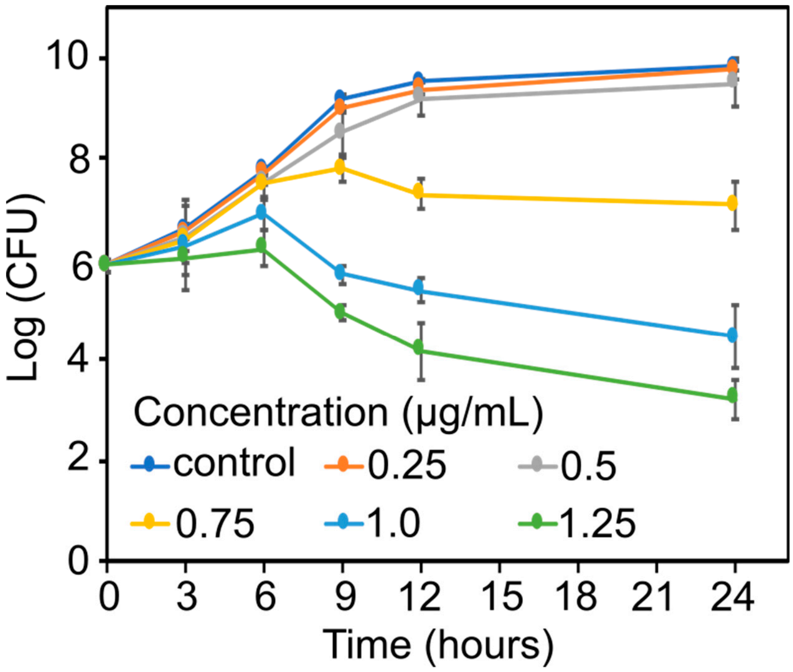

Figure S4 Time-kill curves of $(BiO)_2CO_3$ NPs against PA with various concentrations during the course of 24-h incubation period (mean ± s.d, n =3 replicates)

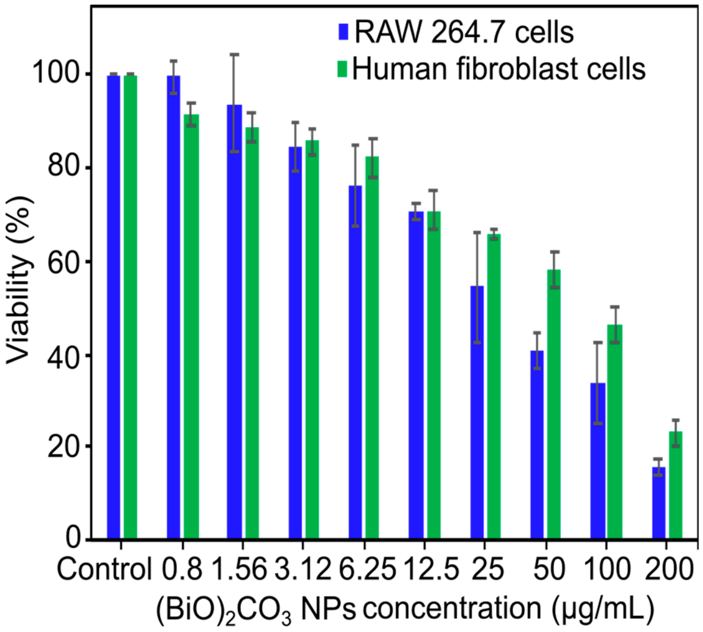

Figure S5 Cell viability curves of $(BiO)_2CO_3$ NPs against human fibroblast cells and murine RAW 264.7 cells

Table S2 FIC index of $(BiO)_2CO_3$ NPs in combination with meropenem antibiotic.

MIC (μg/mL) against DRPA				FIC index
Mero	Mero+ $(BiO)_2CO_3$	$(BiO)_2CO_3$	$(BiO)_2CO_3$ +mero	0.45
2.0	0.25	0.75	0.25	
